# The Resource Conversion Mechanism: Trust, Leader’s Vision of Talent, and Informal Training as Pathways to Organizational Commitment

**DOI:** 10.3390/bs16060944

**Published:** 2026-06-09

**Authors:** Xi Tan, Hyeran Choi, Seung-Wan Kang

**Affiliations:** 1 The Graduate School, Chung-Ang University, Seoul 06974, Republic of Korea; tam0509@cau.ac.kr; 2 College of Business & Economics, Chung-Ang University, Seoul 06974, Republic of Korea; 3 College of Business, Gachon University, Seongnam 13120, Republic of Korea

**Keywords:** trust, leader’s vision of talent, informal training, organizational commitment, generalized structural equation modeling (GSEM)

## Abstract

Organizational commitment is crucial for employee retention and performance; however, little is known about how social and leadership resources translate into organizational commitment through routine learning behaviors. Based on the Conservation of Resources (COR) theory, this study explores how trust and leader’s vision of talent influence organizational commitment through three informal training formats: peer/supervisor coaching, knowledge sharing, and job rotation. Using data from the 2023 Korea Human Capital Enterprise Survey (*N* = 10,371), this study employs a generalized structural equation model that combines Bernoulli logit mediation equations with Gaussian identity outcome equations, along with the bootstrap method, to test the proposed mediation model. The results show that trust and leader’s vision of talent are positively correlated with organizational commitment, whereas knowledge sharing and job rotation significantly mediate these relationships. Peer/supervisor coaching shows no mediating effect. This study conceptualizes informal training as a mechanism through which workplace resources are implemented and translated into employee attitudes, thereby extending COR theory from resource acquisition and protection to resource utilization processes in everyday organizational contexts. The findings suggest that organizations should strengthen trust-based and development-oriented human resource practices to foster employee commitment. These implications extend beyond Korean firms to global HR practitioners seeking to build learning-supportive workplaces.

## 1. Introduction

In contemporary organizations, employees’ organizational commitment has consistently been linked to critical outcomes such as absenteeism, performance, and turnover, making it a central psychological bond underlying organizational effectiveness (Mathieu & Zajac, 1990; Porter et al., 1974). This issue has become particularly important in post-pandemic workplaces, where organizations face changing work arrangements, greater workforce mobility and shifting job demands, and growing demands for continuous learning and development (McPhail et al., 2024; Beier et al., 2025). According to the Conservation of Resources (COR) theory, individuals strive to acquire, maintain, and protect valued resources, particularly social and psychological resources such as guidance and support from supervisors and colleagues. When these resources are sufficient and stable, they foster positive attitudes and behaviors (Hobfoll, 2001).

In this regard, training can be viewed as a crucial mechanism through which organizations acquire and develop resources. This broader perspective is useful for understanding how workplace learning activities shape employee attitudes, including organizational commitment, in organizational settings. Beyond formal, structured programs, employees also learn informally through daily work experiences, interpersonal interactions, and self-directed efforts. Despite decades of investment in formal training programs, up to 70% of workplace learning occurs informally through peer interactions, coaching, and experiential learning (Yoon et al., 2018). In the Korean human resource development (HRD) literature, developmental efforts are not limited to formal classroom-based training. Prior Human Capital Corporate Panel (HCCP)-based research has conceptualized development-related organizational efforts more broadly, including support for organizational change, alignment with business goals, and the provision of developmental opportunities for employees (J. Y. Lee et al., 2018). However, informal training remains understudied as a mechanism linking organizational resources to employee commitment. This gap is particularly important in the Korean context, where hierarchical structures and collectivist norms create unique dynamics in trust building and leadership influence. Prior workplace learning research has distinguished formal and informal training and identified several forms of informal learning, including peer guidance, supervised instruction, self-study, and experiential learning (Lowenstein & Spletzer, 1994). Building on this distinction, this study operationalizes informal training using HCCP items on peer or supervisor coaching, knowledge sharing among employees, or through internal platforms, and job rotation. Studies have shown that informal training constitutes a significant part of the work environment and contributes to employees’ positive attitudes, knowledge and skills acquisition, and performance. Although informal learning is often self-initiated, it is fundamentally embedded in social interactions such as feedback, guidance, coaching, and knowledge exchange with leaders and colleagues (Cerasoli et al., 2017; Yoon et al., 2018; Zajac et al., 2022).

From the COR perspective, trust is a key social and psychological resource. When employees trust their organization and its leaders, they perceive lower resource threats and greater support, thereby developing stronger affective commitment (Celep & Yilmazturk, 2012; Cho & Park, 2011). Similarly, organizational fairness and trust-building leadership behaviors have been found to strengthen commitment (George et al., 2020; C. C. Lee et al., 2022).

A leader’s vision of talent is another crucial resource. A clear and developmental talent vision enhances human resource functions and indirectly influences organizational commitment by increasing employees’ perceived access to growth opportunities (Park & Kim, 2018). Leaders can enhance employees’ capabilities and positive attitudes by demonstrating a vision that supports employee development, fosters shared goals, and promotes informal learning behaviors such as knowledge sharing, thereby further strengthening their loyalty to the organization (AlKayid et al., 2023).

This study uses data from the 2023 HCCP, a nationally representative survey administered by the Korea Research Institute for Vocational Education and Training (KRIVET). The HCCP is a government-authorized panel dataset widely used in organizational and human resource research, which supports its reliability and scholarly relevance.^1^

The 2023 wave remains particularly important in the current context, as it captures Korean firms and employees during the post-pandemic period, when organizations were required to rebuild employee commitment and adapt workplace learning practices to changing work environments. Moreover, as the HCCP survey was not conducted in 2024 due to external circumstances, the 2023 wave is the most recent dataset available for examining these dynamics. For more information about the HCCP, please refer to Appendix A.

Despite the growing body of research on workplace learning and employee attitudes, the existing literature remains fragmented, underscoring the need for the present study. First, although informal training constitutes a substantial share of workplace learning, previous studies have primarily focused on formal training and its outcomes. Consequently, less is known about how informal training contributes to the development of employees’ organizational commitment. Second, because informal training is embedded in everyday interpersonal interactions and resource exchange, social and leadership resources are likely to shape employee engagement in such learning behaviors. However, prior research has paid limited attention to how trust and leader’s vision of talent are translated into informal training behaviors and subsequently, into employee attitudes. In particular, trust has rarely been examined as a core social resource underlying employees’ participation in informal training, and leader’s vision of talent remains insufficiently theorized as a developmental resource that encourages such engagement. Third, although the COR theory has provided important insights into how individuals acquire, protect, and preserve valued resources, less attention has been paid to how these resources are enacted through concrete workplace behaviors. Therefore, the process by which workplace resources are translated into organizational commitment through informal training remains underexplored.

To address these gaps, this study uses large-scale national data from Korean companies to examine how trust and leader’s visions of talent, as social and psychological resources, are associated with employees’ participation in three forms of informal training, which in turn enhance organizational commitment. Drawing on COR theory, we conceptualize trust and leader’s vision of talent as sociopsychological resources that facilitate employees’ engagement in peer or supervisor coaching, knowledge sharing, and job rotation. In doing so, we position informal training not as a peripheral or incidental activity but as a central mechanism through which workplace resources are enacted, utilized, and translated into employee attitudes. By highlighting this resource utilization process, the present study extends COR theory beyond its traditional emphasis on resource acquisition and protection. It advances the understanding of how informal learning serves as a critical pathway linking workplace resources to organizational commitment.

## 2. Theoretical Basis and Hypotheses Development

### 2.1. Organizational Commitment

Organizational commitment refers to the degree to which an employee identifies with and is involved in an organization. According to Porter et al. (1974), organizational commitment consists of three components: (a) identification, which involves adopting the organization’s goals and values as one’s own; (b) involvement, referring to psychological immersion in work-role activities; and (c) loyalty (J. Jang et al., 2019). This type of commitment depends on employees’ acceptance of the organization’s strategy and culture, willingness to work with strong motivation for the organization, and intention to remain with the organization (J. Jang et al., 2019; Porter et al., 1974).

Organizational commitment is considered a key psychological bond for understanding the employee-organization relationship and is frequently used to predict behaviors beneficial to an organization, such as performance, attendance, and retention (Mathieu & Zajac, 1990; Riketta, 2002). A meta-analysis of organizational commitment categorized its primary antecedents into personal characteristics, job characteristics, group and leader relations, organizational characteristics, and role states (Mathieu & Zajac, 1990). Previous studies have shown that various work and leadership factors are significantly associated with commitment. Job and leadership characteristics such as task autonomy, skill diversity, job challenges and scope, leader’s thoughtfulness, effective communication, and participatory leadership have consistently demonstrated significant positive associations with employees’ organizational commitment (Fragkos et al., 2020; Mathieu & Zajac, 1990). Synthetic evidence across occupational groups also suggests that the relationship between commitment and factors, such as work experience and leadership behavior, is generally positive. However, the strength of this relationship varies across occupations (Cohen, 1992). In this study, organizational commitment is conceptualized as the outcome variable. Specifically, we examine how trust and leader’s vision of talent, two critical factors within organizations, relate to organizational commitment and the mechanisms underlying these effects.

### 2.2. Relationships with Trust and the Leader’s Vision of Talent

COR theory posits that resource loss is the principal component of the stress process, while in the context of loss, resource gain becomes particularly important because resources are used both to counteract loss and to prevent further loss. Stress occurs when resources are threatened or lost, or when individuals fail to gain the expected resources after a significant investment. This theory emphasizes that individuals strive to obtain, retain, protect, and foster the resources they value, including objects, conditions, personal characteristics, and energy resources, whose values are both transcultural and shaped by specific cultural contexts. The COR theory highlights the role of the objective nature of the environment and cultural interpretations in the formation of stress, rather than relying solely on individual subjective appraisals (Hobfoll, 2001). Following COR theory, both trust and a leader’s vision of talent can be understood as critical psychological resources that shape employee attitudes. Informal training is the mechanism by which these resources are enacted in daily activities.

According to previous research, trust is an important prerequisite for organizational commitment (George et al., 2020). From the perspective of COR theory, trust can be conceptualized as a critical social and psychological resource. When employees trust their organization and managers, they perceive their resources as less vulnerable to threats and experience greater support and security. Such accumulation and preservation of resources not only reduces anxiety about potential resource loss but also fosters stronger affective commitment. Conversely, when trust is absent, employees are more likely to interpret their circumstances as resource threats, thereby weakening their commitment to the organization (Hobfoll, 2001).

When employees perceive an organization as fair, just, responsible, and trustworthy, they reciprocate with higher levels of organizational commitment (George et al., 2020). Trust exists not only between employees and the organization but also in daily interactions between employees and colleagues. For example, research shows that affective colleague trust and affective organizational commitment jointly moderate the impact of organizational culture on the willingness to share knowledge (K. Y. N. Ng, 2023). In the real estate brokerage industry, leader’s emotional intelligence and leadership behavior can enhance employees’ trust in their supervisors and teams, thereby strengthening their organizational commitment and job performance (C. C. Lee et al., 2022). In education, teachers’ trust in colleagues, managers, and the organization is significantly and positively correlated with multiple dimensions of organizational commitment (Celep & Yilmazturk, 2012). Furthermore, evidence from the public sector shows that trust in senior managers effectively predicts employees’ levels of organizational commitment (Cho & Park, 2011). In conclusion, we argue that trust is a key mechanism through which organizational and interpersonal factors relate to employees’ organizational commitment. Accordingly, we propose the following hypothesis:

**H1.**  *Trust positively relates to organizational commitment.*

Within the COR theory framework, individuals strive to obtain, retain, and protect the resources they value. These resources are not limited to material conditions but also encompass social and psychological support, such as status, security, and development opportunities. When employees perceive these resources as stable and accessible, they are more likely to form positive attitudes and engage in constructive behaviors. Conversely, the loss or threat of resources triggers stress and negative outcomes (Hobfoll, 2001). Thus, leader’s provision of resource-based support and a clear developmental direction serve as critical mechanisms influencing organizational attitudes and behaviors (J. Jang et al., 2019; Kukenberger et al., 2012; Zanabazar et al., 2023). This study focuses on the resource conversion process through which workplace resources are translated into employee attitudes. While the COR theory has primarily emphasized the acquisition, retention, and protection of valued resources (Hobfoll, 2001), the present study argues that mere possession of resources is insufficient to generate organizational commitment. Rather, resources must be actively utilized and converted through concrete workplace behaviors. In this study, trust and leader’s vision of talent represent key relational and leadership resources, whereas peer/supervisor coaching, knowledge sharing, and job rotation function as behavioral pathways through which these resources are converted into organizational commitment.

In the field of organizational commitment, substantial empirical evidence shows that commitment is shaped by a wide range of antecedents, including job and organizational characteristics, leadership, and structural factors (Mathieu & Zajac, 1990). A leader’s clear vision of talent can be regarded as a key resource, providing employees with guidance, recognition, and psychological support (Hobfoll, 2001). Employees who perceive their leader’s vision as clear and supportive are likely to experience greater resource security, which, in turn, enhances their affective bond with the organization (Mathieu & Zajac, 1990; Porter et al., 1974). In this study, leader’s vision of talent refers to employees’ perceptions that senior leaders recognize the importance of selecting, retaining, respecting, and developing talented people, and that they communicate a clear developmental orientation for the organization’s future talent needs. This conceptualization follows Park and Kim’s (2018) HCCP-based work in the Korean context, which highlights leader’s vision of talent as an important organizational factor linked to employee commitment through human resource (HR)-related mechanisms. Building on the empirical findings of Park and Kim (2018), who demonstrated that a leader’s vision of talent has a direct positive effect on HR functions and indirectly influences organizational commitment through HR functions, this study argues that a leader’s vision of talent serves as a pivotal driver for improving HR practices. Enhanced HR functions can expand employees’ access to organizational resources such as support, developmental opportunities, and fair evaluation (Bartlett, 2002; Bulut & Culha, 2010). From the perspective of the COR theory (Hobfoll, 2001), such improvements in HR practices strengthen employees’ perceptions of resource sufficiency and stability, reduce the threat of resource loss, and promote psychological security. Consequently, employees who perceive abundant and well-managed resources within an organization are more likely to develop stronger emotional attachment and commitment (Mathieu & Zajac, 1990; Riketta, 2002). Based on this reasoning, a leader’s vision of talent is expected to be positively related to employees’ organizational commitment. Accordingly, we propose the following hypothesis:

**H2.**  *Leader’s vision of talent positively relates to organizational commitment.*

### 2.3. Informal Training as a Mediator for Trust and Organizational Commitment

Informal training refers to on-the-job learning that does not take place through formal, structured courses but typically unfolds through daily work experience. These include guidance from supervisors or colleagues, self-study using manuals or learning materials, and learning through observation and practice. Informal training is categorized into supervised training (supervisor guidance), peer training (peer guidance), self-study (including manuals and learning materials), and hands-on learning (directly accumulating experience through work) (Lowenstein & Spletzer, 1994). In the Human Capital Enterprise Survey (HCCP) data used in this study, informal training included the following aspects: (A) peer coaching, (B) supervisor coaching, (C) knowledge sharing among employees, (D) knowledge sharing through internal platforms, and (E) learning through job rotation. For the empirical analysis, we further categorized these learning methods into three forms—peer/supervisor coaching, knowledge sharing (among employees and through platforms), and job rotation—and incorporated them into the different dimensions of informal training in our theoretical model. Informal training in an organizational setting typically takes three forms closely related to daily work practices. First, peer or supervisor coaching enables employees to learn through task demonstrations, explanations, and feedback from more experienced colleagues or supervisors during their daily work. It is one of the most common forms of informal workplace training (Lowenstein & Spletzer, 1994). Second, knowledge sharing among employees and across enterprise platforms supports continuous informal training, in which employees improve their performance by collaborating to exchange ideas, solving problems in workplace discussions, and accessing information through digital networks (Taylor & Evans, 2009; X. Zhang & Ren, 2011). Third, new skills acquired due to changes in jobs or responsibilities are considered a form of informal training; therefore, we also include job rotation as a type of informal training (Lowenstein & Spletzer, 1994; Taylor & Evans, 2009). This form is distinct from the other two because it primarily reflects the acquisition of capability resources, allowing employees to broaden their skill sets, expand their role breadth, and enhance their sense of competence through direct task experience.

A growing body of research indicates that informal, experience-based learning is the primary mode of employee development. For example, Yoon et al. (2018) pointed out that, despite significant investments in formal training programs, up to 70% of workplace learning still occurs through informal methods, such as observation and interaction. Similarly, Cerasoli et al. (2017) synthesized the relevant evidence, showing a positive correlation between participation in informal training and key outcomes, including work attitudes, knowledge and skill acquisition, and performance. Informal training has been shown to cultivate positive attitudes, including organizational commitment, job satisfaction, and reduced turnover intention (Bartlett, 2002; Payne & Huffman, 2005; Yoon et al., 2018). Yoon et al. (2018) highlighted that the impact of informal learning on organizational commitment is particularly important because employee commitment largely determines organizational performance. This is because informal learning can enhance employees’ self-efficacy, thereby strengthening their organizational commitment. However, the three forms of informal training are unlikely to influence employees in the same way. Peer/supervisor coaching is more directly tied to interpersonal support and feedback; knowledge sharing is more closely related to the circulation and accumulation of task-relevant knowledge; and job rotation is more strongly associated with experiential skill development and competence expansion. Therefore, distinguishing among these three pathways is theoretically important for understanding how different forms of workplace learning transmit workplace resources into organizational commitment.

From the COR theory perspective, individuals strive to acquire, retain, and protect valuable resources, including social and psychological resources such as guidance, feedback, and recognition from supervisors and colleagues (Hobfoll, 2001). Informal training provides employees with these resources through peer/supervisor coaching, knowledge sharing, and job rotation. It is therefore likely to play a central role in translating relational trust into more positive organizational attitudes. Specifically, trust-based relationships may provide employees with access to guidance, information, and developmental opportunities; however, these resources are unlikely to automatically influence organizational commitment. Instead, they are expected to be translated into commitment through concrete behavioral processes, such as peer/supervisor coaching, knowledge sharing, and job rotation. Through coaching, employees receive relational support and feedback, through knowledge sharing, they gain access to cognitive and informational resources, and through job rotation, they accumulate broader capabilities and task-related competencies. Previous research has shown that organizational support for informal training (e.g., opportunities for on-the-job training, coaching, mentoring, and practice communities) not only improves learning efficiency and reduces costs but also enhances performance and strengthens employees’ organizational commitment (Cerasoli et al., 2017; Yoon et al., 2018). Based on this, broader research on training support shows a positive correlation between developmental support from supervisors and colleagues and organizational commitment, which is often manifested in employees’ perceived organizational support (Bartlett, 2002; Bulut & Culha, 2010; Cho & Park, 2011).

Based on this, we anticipate that informal training will constitute a key mechanism through which employees’ trust in their supervisors and colleagues translates into organizational commitment. Specifically, peer/supervisor coaching, knowledge sharing (among employees and through internal platforms), and job rotation are considered different forms of informal training that can transfer social and psychological resources from trust relationships, thereby enhancing employees’ self-efficacy and competence and strengthening organizational commitment, leading to the following hypotheses. At the same time, because peer/supervisor coaching, knowledge sharing, and job rotation represent distinct resource conversion pathways, they are examined separately rather than treated as a single undifferentiated construct. Accordingly, the following hypotheses are proposed:

**H3.**  *Informal training mediates the relationship between trust and organizational commitment.*

**H3a.**  *Peer/supervisor coaching mediates the relationship between trust and organizational commitment.*

**H3b.**  *Knowledge sharing mediates the relationship between trust and organizational commitment.*

**H3c.**  *Job rotation mediates the relationship between trust and organizational commitment.*

### 2.4. Informal Training as a Mediator for the Leader’s Vision of Talent and Organizational Commitment

Within the COR theory framework, individuals strive to obtain, retain, and protect valued resources; supervisor understanding, coworker support, and progress through education or work training are regarded as critical conditional and energy resources, whose acquisition and maintenance foster positive attitudes and behaviors (Hobfoll, 2001). A leader’s clear vision of talent provides employees with important psychological and social resources that signal recognition, support, and development opportunities (AlKayid et al., 2023). These resources strengthen employees’ motivation to participate in developmental activities, particularly informal training outside formal curricula through peer or supervisor coaching, knowledge sharing among colleagues, or through digital platforms, and job rotation (Cerasoli et al., 2017; Lowenstein & Spletzer, 1994; Yoon et al., 2018).

A leader’s vision of talent is likely to promote the three forms of informal training differently. Specifically, a clear talent vision may foster peer/supervisor coaching by signaling that feedback and guidance are meaningful investments in employee development, encourage knowledge sharing by emphasizing that collaboration and expertise exchange are valued, and support job rotation by legitimizing broader developmental opportunities and cross-functional growth. In this sense, peer/supervisor coaching, knowledge sharing, and job rotation represent distinct pathways through which a leader’s developmental vision can be translated into employees’ learning experiences, and ultimately, organizational commitment. Informal training serves as a critical mediating mechanism, translating the resources provided by the leader into concrete experiences that reinforce employees’ skills, knowledge, and supportive relationships. Employees who perceive a clear talent vision from their leaders are more likely to participate in informal training, thereby accumulating resources to strengthen their sense of competence and belonging. In turn, these experiences enhance their affective bonds with the organization and are positively associated with organizational commitment. Empirical evidence further supports the role of informal training as a channel through which leadership and support relate to commitment, demonstrating that on-the-job guidance, knowledge sharing, and experiential learning contribute significantly to employee development and organizational outcomes (Bartlett, 2002; Bulut & Culha, 2010; Kittel & Seufert, 2023; Lowenstein & Spletzer, 1994; X. Zhang & Ren, 2011).

Based on this reasoning, informal training is proposed as a mediator between a leader’s vision of talent and organizational commitment. Peer/supervisor coaching, knowledge sharing, and job rotation are distinct forms of informal training that facilitate this mediating process, leading to the following hypotheses:

**H4.**  *Informal training mediates the relationship between the leader’s vision and organizational commitment.*

**H4a.**  *Peer/supervisor coaching mediates the relationship between the leader’s vision of talent and organizational commitment.*

**H4b.**  *Knowledge sharing mediates the relationship between the leader’s vision of talent and organizational commitment.*

**H4c.**  *Job rotation mediates the relationship between the leader’s vision of talent and organizational commitment.*

### 2.5. Informal Training as a Resource Conversion Mechanism

COR theory posits that employees strive to acquire, protect, and invest valued resources and that existing social and psychological resources must be actively transformed into usable capabilities to prevent potential resource loss and promote resource gain (Hobfoll, 2001). Building on this logic, this study views informal training as a COR-based resource-conversion mechanism, through which relational and leadership resources are behaviorally enacted before shaping employee attitudes. Workplace learning research emphasizes that informal training is inherently embedded in daily work and is influenced by interaction, problem-solving, and the broader social environment, rather than structured instruction. As Marsick and Watkins (2015) argue, informal learning stems from routine work activities such as coaching, mentoring, performance planning, and trial-and-error experiments, which enable employees to continuously reinterpret work situations and develop their own capabilities. Prior research emphasizes that informal learning is socially constructed, implicit, and often triggered by work challenges, making it a key mechanism through which employees acquire, reorganize, and apply knowledge in real time (Marsick et al., 2017). Therefore, informal learning is a dynamic process that arises in complex work environments, where individuals must adapt quickly, collaborate with others, and cope with uncertainty. Under such conditions, social communication and cross-boundary interaction can accelerate learning (Watkins & Marsick, 2023). Taken together, these insights suggest that trust and the leader’s vision of talent do not automatically influence organizational commitment. Rather, when employees feel trusted and receive developmental guidance from leaders, these relational and leadership resources may encourage them to engage in informal training activities such as coaching, knowledge sharing, and job rotation. Through these behavioral pathways, informal training helps transform interpersonal and leadership resources into enhanced competence, confidence, and belonging, thereby strengthening employees’ emotional organizational commitment (Marsick & Watkins, 2015; Marsick et al., 2017; Watkins & Marsick, 2023).

**Figure 1:**
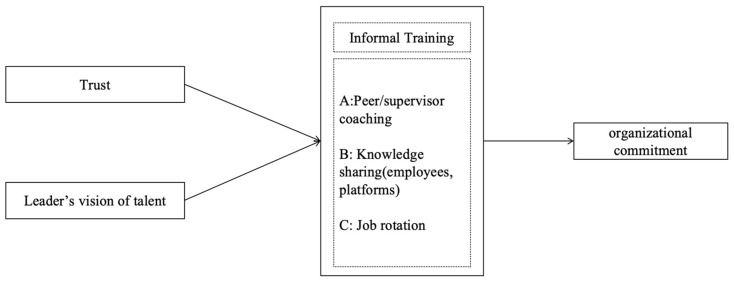
Theoretical Model.

An overview of the proposed theoretical framework is presented in Figure 1.

## 3. Method

### 3.1. Procedure and Respondents

After excluding respondents aged 60 and older and those under 20 years, the final sample for the fourth round of surveys (2023) included 10,371 employees. Regarding gender distribution, 7246 male respondents (69.9%) and 3125 female respondents (30.1%) were included. Regarding educational level, 0.8% (85 people) had a high school diploma or lower, 22.6% (2345 people) were high school graduates, 17.2% (1782 people) had completed associate’s degrees, 55.3% (5738 people) held bachelor’s degrees, and 4.1% (421 people) held postgraduate degrees. In terms of company size, 66.2% (6867 respondents) were employed by small companies with 100–299 employees, 24.0% (2488 people) by medium-sized companies with 300–999 employees, and 9.8% (1016 people) by large companies with more than 1000 employees. Industry distribution showed that the vast majority of employees worked in manufacturing (8270 people, 79.74%), followed by non-financial services (1694 people, 16.33%) and financial services (407 people, 3.92%).

### 3.2. Measures

As this study relies on secondary survey data from the HCCP, the operationalization of key variables is necessarily bounded by the original questionnaire items. The HCCP is a large-scale survey developed and administered by KRIVET. It has been widely used to examine human resource development and organizational practices in Korean firms. Following prior HCCP-based research, this study adopts cautious labels that reflect the substantive content of the available measures, rather than assuming perfect equivalence with all related constructs in the broader literature (J. Y. Lee et al., 2018).

Informal training is defined as the acquisition of knowledge and skills through informal means at work (Lowenstein & Spletzer, 1994). While this study adopts Lowenstein and Spletzer’s (1994) conceptual framework to distinguish formal and informal learning, the empirical operationalization is based on items drawn from the HCCP survey. In this study, the HCCP items were operationalized as three forms of informal training. Specifically, the HCCP measures five workplace developmental activities: (A) peer coaching and guidance (W23Q15A1), (B) supervisor coaching and guidance (W23Q15B1), (C) knowledge sharing among employees (W23Q15C1), (D) knowledge sharing through internal platforms (W23Q15D1), and (E) learning through job rotation (W23Q15E1). For the empirical analysis, these activities were grouped into three dummy variables (1 = participation, 0 = non-participation): peer/supervisor coaching (A and B), knowledge sharing (C and D), and job rotation (E). The remaining three core variables—trust, leader’s vision of talent, and organizational commitment—were measured using nine items on a 5-point Likert scale (1 = strongly disagree, 5 = strongly agree). The Cronbach’s alpha coefficients for the measurement scales ranged from 0.752 to 0.925, indicating satisfactory internal consistency.

Organizational commitment was measured using three items. Examples include “I feel this company’s problems are my own.” If I decided to leave this company, I would lose too much of my life.” Cronbach’s alpha for organizational commitment was 0.788. In this study, organizational commitment was operationalized as a general HCCP-based commitment construct rather than as a unidimensional measure of affective commitment. Given that the item set contained elements associated with both affective attachment and continuance considerations, the construct should be interpreted as reflecting multiple dimensions of organizational commitment.

Leader’s vision of talent was measured using four HCCP items that capture employees’ perceptions of management’s talent development orientation. Sample items include “Our company rewards outstanding talent” and “Our company’s management emphasizes the importance of talent whenever possible.” This operationalization follows prior HCCP-based research in Korea, which conceptualized leader’s vision of talent as senior leader’s orientation toward selecting, retaining, respecting, and developing talented employees (Park & Kim, 2018). Cronbach’s alpha for this measure was 0.918.

Trust was measured using two HCCP items: “In our company, colleagues trust each other” and “Our company’s management is trustworthy and reliable in every aspect.” As these items capture perceptions of trust toward coworkers and management, this study interprets trust as a broad perception of workplace trust. Cronbach’s alpha for this measure was 0.731.

All reliability coefficients exceeded the recommended threshold of 0.70, indicating satisfactory internal consistency.

### 3.3. Control Variables

Consistent with prior research (T. W. Ng & Feldman, 2010), demographic variables (gender, age, and education) and organizational context variables (firm size and industry) were included as controls, as they may relate to both participation in informal training and levels of organizational commitment.

### 3.4. Statistical Analysis

The statistical analyses were performed in several steps. Descriptive statistics and correlation analyses were performed to examine the basic characteristics of the study variables. Second, reliability analysis was conducted to assess the internal consistency of the measurement items, with coefficients above 0.70 considered acceptable according to conventional psychometric guidelines (Nunnally & Bernstein, 1994). Third, a confirmatory factor analysis (CFA) was conducted to evaluate the construct validity of the measurement model. Model fit was assessed using multiple fit indices, including the chi-square statistic, root mean square error of approximation (RMSEA), comparative fit index (CFI), Tucker–Lewis index (TLI), and standardized root mean square residual (SRMR). Following commonly used guidelines, RMSEA values below 0.08 were considered acceptable and values below 0.06 indicated good fit; CFI and TLI values above 0.90 indicated acceptable fit and values above 0.95 indicated good fit; and SRMR values below 0.08 indicated acceptable fit (Browne & Cudeck, 1993; Hu & Bentler, 1999; Kline, 2023). Finally, structural equation modeling was used to test the hypothesized relationships between trust, leader’s vision of talent, informal training, and organizational commitment, including the mediating role of informal training.

## 4. Results

Stata 18.0 was used for all statistical analyses (StataCorp, 2023c). First, an ordinary least squares regression was conducted to examine the direct effects. Next, because the mediation models included binary mediators and a continuous outcome, generalized structural equation modeling (GSEM) was employed to allow for different family-link specifications within a single framework (Muthén & Asparouhov, 2015; StataCorp, 2023b). Specifically, informal training mediators were estimated using a Bernoulli family with a logit link. In contrast, organizational commitment was modeled as a continuous outcome under a Gaussian family with an identity link (StataCorp, 2023b). In addition, the indirect effects were assessed using bootstrap resampling with 5000 replications because the indirect effects were computed as functions of the estimated coefficients and their sampling distributions may be non-normal (Muthén & Asparouhov, 2015; StataCorp, 2023a). Finally, the variance inflation factor values for all variables were below five, indicating that multicollinearity was not a serious concern.

### 4.1. Confirmatory Factor Analysis

After excluding the binary mediating variable, a CFA was conducted on the remaining three core latent constructs. The results indicated that the three-factor measurement model exhibited a good fit to the data, with RMSEA = 0.054, CFI = 0.987, TLI = 0.981, and SRMR = 0.021. In addition, all standardized factor loadings were statistically significant, ranging from 0.660 to 0.903, thereby demonstrating that the measurement items had strong explanatory power for their corresponding latent constructs. Furthermore, the average variance extracted values for all three constructs exceeded 0.50, and the composite reliability values were above 0.70, suggesting satisfactory convergent validity and internal consistency. Overall, the measurement quality of the three core variables used in this study was adequate and suitable for the subsequent hypothesis testing.

### 4.2. Correlation Analysis

After confirming the absence of multicollinearity, we conducted a correlation analysis. As shown in Table 1, the correlation analysis revealed significant positive relationships between the study variables. Leader’s vision of talent was positively correlated with organizational commitment (*r* = 0.489, *p* < 0.001) and trust (*r* = 0.727, *p* < 0.001). Moreover, “Trust” was significantly correlated with organizational commitment (*r* = 0.513, *p* < 0.001). These significant positive correlations provide preliminary support for our hypotheses, which we formally test using a regression analysis in the next section. We assessed discriminant validity using the average variance extracted (AVE) (Fornell & Larcker, 1981). The square root of the AVE for each factor exceeded the corresponding correlation coefficients, indicating adequate discriminant validity of the variables.

### 4.3. Hypothesis Testing

As shown in Table 2, the unified GSEM results revealed that trust had a significant positive direct effect on organizational commitment (*B* = 0.324, *p* < 0.001). Similarly, a leader’s vision of talent exerted a significant positive direct effect on organizational commitment (*B* = 0.213, *p* < 0.001). Thus, H1 and H2 were supported.

Moreover, as shown in Table 2, the unified GSEM results revealed that trust was positively and significantly associated with all three informal training pathways: peer/supervisor coaching (*B* = 0.469, *p* < 0.001), knowledge sharing (*B* = 0.393, *p* < 0.001), and job rotation (*B* = 0.403, *p* < 0.001). Similarly, leader’s talent visions exerted significant positive effects on peer/supervisor coaching (*B* = 0.129, *p* < 0.001), knowledge sharing (*B* = 0.526, *p* < 0.001), and job rotation (*B* = 0.352, *p* < 0.001). These results indicate that both relational and leadership-based resources play important roles in promoting employees’ involvement in informal training processes.

To examine the mediating role of informal training in the relationships between trust, leader’s vision of talent, and organizational commitment, this study employed a bootstrap procedure based on the unified GSEM model. Because the mediators were modeled as binary variables with logit links, caution is warranted in interpreting the resulting indirect effects. Specifically, these estimates are derived from products of coefficients obtained on different metrics, combining logit-scale coefficients for categorical mediators with linear-scale coefficients for organizational commitment. Prior methodological research has noted that, when categorical mediators or outcomes are involved, indirect effects cannot be interpreted in the same way as the product of coefficient estimates in conventional linear mediation models (Muthén & Asparouhov, 2015). Therefore, the present study interprets these estimates primarily as indicators of the presence and statistical significance of indirect pathways rather than as strictly comparable magnitudes across alternative mediation channels.

**Figure 2:**
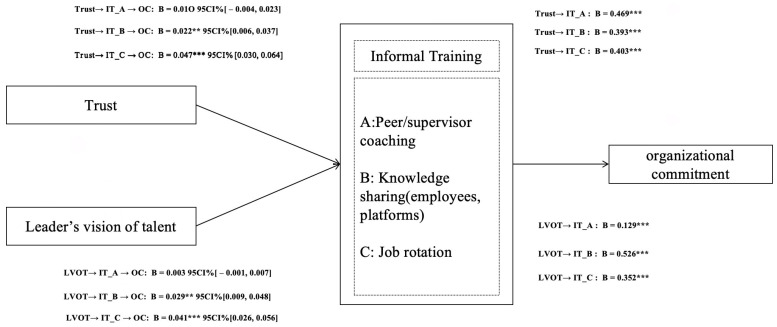
Final Structural Model with Estimated Path Coefficients. ** *p* < 0.01, *** *p* < 0.001.

As shown in Table 3, trust exerted significant positive indirect effects on organizational commitment through knowledge sharing (Effect = 0.022, SE = 0.008, 95% CI [0.006, 0.037]) and job rotation (Effect = 0.047, SE = 0.009, 95% CI [0.030, 0.064]), whereas the indirect effect through peer/supervisor coaching was not significant (Effect = 0.010, SE = 0.007, 95% CI [−0.004, 0.023]). The total indirect effect of trust was significant (Effect = 0.078, SE = 0.010, 95% CI [0.058, 0.098]). Leader’s vision of talent showed significant positive indirect effects on organizational commitment through knowledge sharing (Effect = 0.029, SE = 0.010, 95% CI [0.009, 0.048]) and job rotation (Effect = 0.041, SE = 0.008, 95% CI [0.026, 0.056]) but not through peer/supervisor coaching (Effect = 0.003, SE = 0.002, 95% CI [−0.001, 0.007]). The total indirect effect of leader’s vision of talent was also significant (Effect = 0.072, SE = 0.010, 95% CI [0.052, 0.092]). Taken together, these findings suggest that the mediating effects of trust and leader’s vision of talent primarily operated through knowledge sharing and job rotation rather than through coaching by peers and supervisors. Therefore, H3b, H3c, H4b, and H4c were supported, whereas H3a and H4a were not. All the estimated path coefficients are presented in Figure 2.

Although trust and leader’s vision of talent remained significant when entered simultaneously into the unified GSEM model, their relatively high correlation suggests caution is warranted in interpreting their unique effects. In particular, the distinct contribution of a leader’s vision of talent may be less clearly separable from trust in some pathways, such as peer/supervisor coaching.

## 5. Discussion

This study examines how trust and a leader’s vision of talent relate to organizational commitment through informal training within the framework of COR theory (Hobfoll, 2001). Our findings have several important implications for future studies. First, both trust and a leader’s vision of talent were found to have significant positive effects on employees’ participation in informal training, indicating that relational and leadership resources can encourage workplace learning behaviors. From the COR perspective, this finding suggests that employees are more likely to invest effort in informal learning when they perceive sufficient social support, developmental guidance, and resource availability in the workplace. Second, knowledge sharing and job rotation mediated the relationships between these antecedent variables and organizational commitment, whereas peer/supervisor coaching did not. These results suggest that trust and leader’s vision of talent contribute not only directly but also indirectly to organizational commitment by promoting employees’ engagement in specific forms of informal training. Knowledge sharing may expand employees’ access to task-related knowledge and social connections, whereas job rotation may provide broader developmental experiences and opportunities to gain resources (Peng, 2024; B. Zhang et al., 2025). Taken together, these findings support the proposed model and underscore the importance of informal training as a key mechanism that links valuable organizational resources to positive employee attitudes. Simultaneously, the results also suggest that not all forms of informal training function equally in translating workplace resources into organizational commitment, highlighting the need to distinguish between different informal learning activities rather than treating informal training as a single uniform process.

### 5.1. Key Findings

This study examines how social and psychological resources enhance organizational commitment through informal training. The findings generally support the core proposition of the COR theory, namely that employees seek to acquire, invest in, and retain valuable resources to protect their well-being and sustain positive work attitudes (Hobfoll, 2001). Specifically, the results reveal significant mediation pathways linking trust and a leader’s vision of talent to organizational commitment through knowledge sharing and job rotation, whereas peer/supervisor coaching did not show a significant mediating effect. This interpretation may be particularly relevant in the Korean organizational context, in which hierarchical relationships, power distance, and seniority-based norms shape how employees interpret interactions with supervisors or senior colleagues. Viewed through the lens of power distance, employees in relatively hierarchical organizational cultures may interpret supervisor-related interactions not only as developmental support but also as signals of authority, evaluation, or control (Hofstede, 2001). Recent studies on Korean workplaces also suggest that hierarchical and power-distance dynamics can influence interpersonal relationships, open communication, and employees’ willingness to express opinions or disclose concerns (H. Jang et al., 2025; Eon Sim & Jang, 2025). In this context, peer or supervisor coaching may not always be perceived purely as a resource-gaining developmental opportunity. Rather, when such guidance is embedded in seniority-based or authority-laden relationships, employees may interpret it as monitoring, correction, or an additional interpersonal obligation. From the COR perspective, this may weaken the role of coaching as a resource-gain mechanism and even turn it into a potential source of resource depletion. These findings suggest that informal training should not be treated as a uniform construct because different forms of informal learning may translate organizational resources into different positive employee outcomes. Overall, the findings highlight the importance of trust, leadership support, and differentiated forms of informal training in explaining how employees develop a stronger attachment to their organization.

### 5.2. Theoretical Implications

#### 5.2.1. Extending COR Theory: Informal Training as a Resource Conversion Mechanism

This study contributes to the COR theory by shifting attention from the mere possession of resources to the process through which resources are behaviorally utilized and translated into employee attitudes. Prior COR research has been highly influential in explaining how individuals seek to acquire, retain, and protect valued resources and how resource gains and losses shape motivation and well-being. However, compared with these themes, less explicit attention has been paid to the workplace mechanisms through which relational and leadership-based resources are enacted in everyday work and subsequently converted into attitudinal outcomes. In particular, existing COR-informed studies have often assumed that beneficial resources will naturally lead to positive employee outcomes, without sufficiently specifying the behavioral processes through which such effects materialize. The present findings help address this gap by showing that trust and a leader’s vision of talent are not beneficial in a direct sense; rather, their effects are partly translated into organizational commitment through employees’ participation in informal training. More importantly, our findings indicate that the conversion process is not uniform across all forms of informal training. Knowledge sharing and job rotation emerged as meaningful mediating pathways, whereas peer/supervisor coaching did not have a significant indirect effect. This pattern suggests that the positive effects of relational and leadership resources do not automatically unfold through learning-related behaviors. Instead, when employees engage in learning processes that expand task-relevant knowledge and developmental capacity, these factors appear to be converted more effectively into commitment.

These findings extend the COR theory in two ways. First, they show that workplace resources should be understood not only in terms of acquisition and protection but also in terms of their enactment in everyday organizational behaviors. In this study, informal training represents a key channel through which employees utilize relational and developmental resources. Second, the findings suggest that various forms of informal training operate differently during the process. Knowledge sharing and job rotation appear to translate resources into organizational commitment by expanding employees’ access to expertise, skills, and developmental experiences. In contrast, peer/supervisor coaching may not always function as a resource-gaining process, particularly in hierarchical organizational contexts, where it can be perceived as evaluation or pressure. In this way, the study refines the COR theory by showing that the attitudinal value of resources depends not only on their availability but also on the specific behavioral channels through which they are experienced and interpreted.

#### 5.2.2. Reconciling Trust and Leadership Research

This study also contributes to the literature by integrating two lines of research that are often developed separately: trust- and leadership-based developmental research. Prior studies have consistently shown that trust and supportive leadership are both positively associated with employee attitudes, including organizational commitment. However, these research streams typically treat trust and leadership as parallel antecedents, offering limited explanations of how their effects are realized through employees’ everyday developmental experiences. Consequently, the mechanisms linking social relationships, leadership signals, and employee commitment remain fragmented.

The present study helps reconcile these research streams by showing that trust and a leader’s vision of talent operate as distinct but complementary workplace resources, whose effects are partly transmitted through informal training. Trust provides a relational foundation that reduces uncertainty and facilitates openness to interpersonal exchanges, while a leader’s vision of talent signals to employees that they are valued and worthy of future growth. Examining these two resources within the same framework reveals that organizational commitment is shaped not only by employees’ sense of relational safety but also by their perception that leaders recognize and invest in their developmental potential. Simultaneously, the results indicate that the two resources do not operate in parallel; they become meaningful partly through employees’ engagement in informal learning behaviors. This integrative perspective advances the theory by identifying informal training as a bridge between social resources and attitudinal outcomes. Rather than treating trust, leadership support, and training as separate predictors of commitment, this study demonstrates how they are interconnected in a broader developmental process. In particular, knowledge sharing and job rotation appear to be more consequential channels through which trust and a leader’s vision of talent are translated into organizational commitment. This finding adds nuance to both trust and leadership research by suggesting that the value of these resources depends not only on their presence but also on whether employees are embedded in learning processes that allow those resources to be enacted and accumulated over time.

Concurrently, this study adopts a broad workplace trust perspective rather than a narrowly differentiated trust construct. As the HCCP items capture employees’ trust perceptions of coworkers and management, trust is interpreted here as a generalized relational resource embedded in the overall workplace context. Accordingly, the findings should be understood as reflecting the broader trust climate of the organization rather than the isolated effects of coworker trust and management trust as separate constructs.

### 5.3. Practical Implications

The findings offer practical guidance for organizations seeking to enhance their organizational commitment through informal training. Managers should focus on building trust-based relationships to ensure fairness, consistency, and transparency. A high-trust environment enables employees to engage more actively in informal training without fear of negative evaluations or excessive competition. Second, leaders should communicate their vision of talent and emphasize their long-term developmental goals. When employees understand how their personal growth aligns with organizational objectives, they are more likely to participate in informal training activities, such as coaching, knowledge sharing, and job rotation. Third, organizations should establish systems that support informal training. For example, coaching programs, digital knowledge-sharing platforms, and cross-departmental job rotation schemes can facilitate continuous learning and development. Such initiatives can strengthen not only employees’ skills but also their sense of belonging and value within the organization (Booth, 1991; Zajac et al., 2022). Finally, organizations should ensure that informal training takes place within an ethical and transparent culture. Johnson et al. (2018) suggested that social resources such as cohesion and peer influence may produce unintended consequences if they are not properly guided. Therefore, firms should embed ethical norms and integrity systems into learning and development practices.

This study, which is grounded in the organizational context of South Korea, also provides important cultural and contextual insights into the proposed mechanism. South Korean organizations are often characterized by collectivistic values and strong relational norms, in which trust plays a central role in collaboration, and leaders are expected to take an active role in employee development. In this context, trust and the leader’s vision of talent may become especially important in motivating employees to engage in informal training and strengthen their organizational commitment. These social and psychological resources are likely to be particularly salient in close-knit organizational environments, where interpersonal relationships and developmental support are highly valued. Accordingly, the findings suggest that the effects of trust, leadership, and informal training should be understood not only as general organizational mechanisms but also as processes shaped by the broader cultural context in which employees work.

### 5.4. Limitations and Future Research

First, the cross-sectional design limits causal inferences. Although these findings provide meaningful evidence for the proposed relationships, future studies should employ longitudinal or multilevel designs to examine how resource accumulation and conversion through informal training develop over time.

Second, this study focused on Korean firms, which are characterized by hierarchical and collectivist structures. Future research could examine whether the relationships among trust, informal training, and organizational commitment hold in more individualistic or egalitarian cultures.

Third, this study has measurement-related limitations concerning both informal training and trust. Specifically, informal training was measured using a set of binary indicators reflecting whether employees participated in peer or supervisor coaching, knowledge sharing, and job rotation. While this operationalization is useful for identifying participation patterns, it does not capture important differences in the frequency, intensity, duration, or quality of these learning experiences. Therefore, the findings should be interpreted as evidence of participation-based effects, rather than the full developmental impact of informal training. In addition, trust was measured using items referring to both coworkers and management, which may limit the construct specificity. Accordingly, the trust variable should be interpreted as reflecting broad workplace trust, rather than distinct forms of target-specific trust. Future research should adopt more fine-grained measures to examine how the depth and quality of informal training, as well as differentiated forms of trust, shape organizational commitment and related employee outcomes.

Fourth, although the questionnaire data were derived from employee self-reports in the HCCP, the correlation coefficients between the main variables did not exceed the recommended threshold of 0.75. This indicates that these measures have acceptable discriminant validity and that the core constructs can be empirically distinguished. However, future research could further enhance construct validity by incorporating additional measurement methods, such as multi-source data, behavioral indicators, or supervisor-reported assessments, thereby improving the robustness of discriminant validity and reducing reliance on self-reported measures.

Fifth, because this study relied on self-reported data, common method bias (CMB) could not be completely ruled out. To mitigate this limitation, future research could incorporate evaluations of supervisor reports, employ multi-source or multilevel designs, or utilize a longitudinal analysis across multiple rounds of data from the HCCP survey to further reduce the potential risks associated with CMB.

Sixth, this study did not fully examine differences across industries or work contexts. Employees in manufacturing, non-financial services, and financial services may experience different task structures, skill requirements, learning opportunities, and leadership practices. For example, manufacturing firms may emphasize standardized skills, process efficiency, and technical routines. In contrast, service and financial firms may place greater emphasis on customer interactions, professional expertise, compliance, and relational capabilities. Such differences may influence how employees interpret the leader’s vision of talent, developmental guidance, job rotation, and informal learning opportunities. Therefore, the meaning and effectiveness of developmental leadership resources may vary depending on industry-specific work conditions. Future research could compare industry-specific patterns or test whether industry context moderates the relationships among leader’s vision of talent, informal training, and organizational commitment.

Finally, future research could test potential moderating factors, such as organizational climate or leadership ethics, to clarify when and under what conditions trust and a leader’s vision of talent are most strongly associated with informal training and organizational commitment.

## 6. Conclusions

In summary, this study provides empirical support for a resource-based model of organizational commitment. Integrating trust, a leader’s vision of talent, and informal training within the framework of COR theory demonstrates how social and psychological resources are transformed into enduring organizational commitment through workplace learning processes. The findings deepen the theoretical understanding of the resource–learning–commitment pathway and offer practical insights for organizations seeking to build trust-based and learning-oriented environments that sustain long-term employee engagement.

## Figures and Tables

**Table 1:** Correlation Analysis.

Variables	Mean	SD	(1)	(2)	(3)
1. Leader’s vision of talent	3.181	0.849	(0.860)		
2. Organizational commitment	2.980	0.772	0.489 ***	(0.747)	
3. Trust	3.339	0.779	0.727 ***	0.513 ***	(0.775)

**Note.** The square root of the AVE is reported along the diagonal. Because listing all control variables would lengthen the table, they are not reported here. They are available from the authors upon request. *** *p* < 0.001.

**Table 2:** Direct Effects from the Unified GSEM Model.

Predictor Path	*B*	SE	95% CI
Trust → Peer/supervisor coaching	0.469 ***	0.039	[0.391, 0.546]
Trust → Knowledge sharing	0.393 ***	0.046	[0.303, 0.483]
Trust → Job rotation	0.403 ***	0.043	[0.318, 0.488]
LVOT → Peer/supervisor coaching	0.129 ***	0.035	[0.060, 0.198]
LVOT → Knowledge sharing	0.526 ***	0.043	[0.442, 0.611]
LVOT → Job rotation	0.352 ***	0.040	[0.273, 0.431]
Peer/supervisor coaching → OC	0.021	0.015	[−0.008, 0.050]
Knowledge sharing → OC	0.055 **	0.019	[0.018, 0.091]
Job rotation → OC	0.116 ***	0.017	[0.082, 0.150]
Trust → OC	0.324 ***	0.013	[0.298, 0.349]
LVOT → OC	0.213 ***	0.012	[0.189, 0.237]

**Note.** Unstandardized coefficients are reported. The three informal training mediators were estimated using logit links, whereas organizational commitment was estimated using an identity link. LVOT = Leader’s vision of talent; OC = Organizational commitment. ** *p* < 0.01, *** *p* < 0.001. Control variables were included in all equations but are omitted from the table for brevity.

**Table 3:** Indirect Effects from the Unified GSEM Model.

Indirect Path	Effect	Bootstrap SE	95% CI
Trust → Peer/supervisor coaching → OC	0.010	0.007	[−0.004, 0.023]
Trust → Knowledge sharing → OC	0.022 **	0.008	[0.006, 0.037]
Trust → Job rotation → OC	0.047 ***	0.009	[0.030, 0.064]
Total indirect effect (trust)	0.078 ***	0.010	[0.058, 0.098]
LVOT → Peer/supervisor coaching → OC	0.003	0.002	[−0.001, 0.007]
LVOT → Knowledge sharing → OC	0.029 **	0.010	[0.009, 0.048]
LVOT → Job rotation → OC	0.041 ***	0.008	[0.026, 0.056]
Total indirect effect (LVOT)	0.072 ***	0.010	[0.052, 0.092]

**Note.** Unstandardized indirect effects are reported. Indirect effects were estimated from the unified GSEM model using 5000 bootstrap replications. Control variables were included in all equations. LVOT = leader’s vision of talent; OC = organizational commitment. ** *p* < 0.01, *** *p* < 0.001.

**Figure A1:**
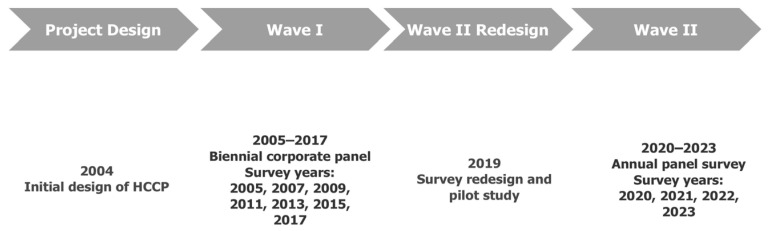
Survey History and Panel Structure of HCCP.

**Figure A2:**
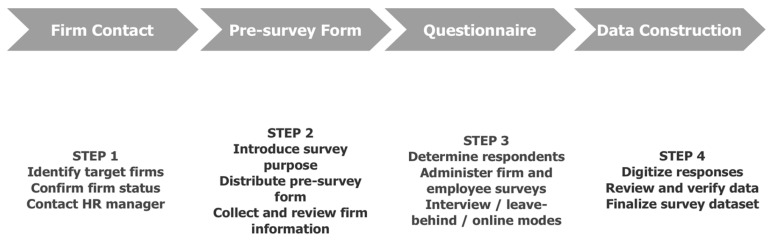
Main Stages of the HCCP Data Collection Procedure.

**Figure A3:**
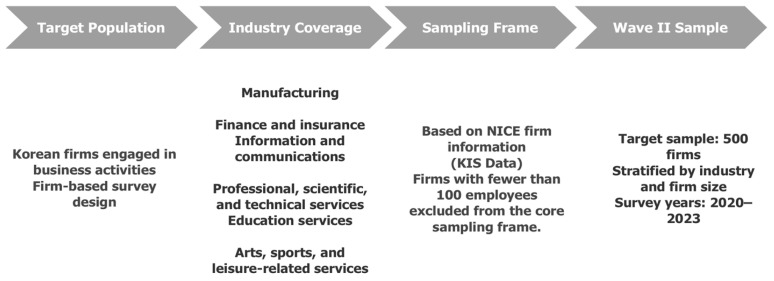
Target Population, Industry Coverage, and Sample Design of HCCP Wave II.

## Data Availability

The data are publicly accessible and can be obtained directly from the official website of the Korea Research Institute for Vocational Education and Training (KRIVET). https://www.krivet.re.kr/kor/sub.do?menuSn=202 (accessed on 27 May 2026).

## Note

1 The HCCP is a nationally organized survey approved by Statistics Korea (Approval No. 389003). The panel has developed through two phases: the first phase was conducted biennially, whereas the second phase, launched in 2019, operates as an annual survey. This structure reflects the continuity and institutional stability of the dataset. Prior studies have also utilized HCCP data across various organizational and HR-related topics. For example, Park and Kim (2018) examined the roles of organizational culture, a leader’s vision of talent, and training in shaping employee commitment, while Kang et al. (2018) investigated the replacement costs of turnover in Korea.
